# Dataset of human platelets in healthy and individuals with cardiovascular pathology obtained by surface-enhanced Raman spectroscopy

**DOI:** 10.1016/j.dib.2020.105145

**Published:** 2020-01-15

**Authors:** Andrey Zyubin, Vladimir Rafalskiy, Anna Tcibulnikova, Karina Matveeva, Ekaterina Moiseeva, Alina Tsapkova, Ilia Samusev, Valery Bryukhanov, Maksim Demin

**Affiliations:** REC «Fundamental and Applied Photonics. Nanophotonics», Immanuel Kant Baltic Federal University, A.Nevskogo St. 14, Kaliningrad, Russia

**Keywords:** Raman spectroscopy, Platelet, SERS, Gold nanoparticles, Aminoacids

## Abstract

This data article contains Raman experimental data, obtained with Centaur U Raman spectrometer (Russia), which can be used for rapid and early structure changes and biomarkers identification in individuals with cardiovascular decease (CVD) pathology *in vitro*. The data include analyzed Surface-Enhanced Raman Scattering (SERS) spectra of human platelets taken from healthy individuals and individuals with cardiovascular pathology. Data can provide information about characteristic maxima of different cell components and its changes in platelets.

**Table:** Specifications Table

Subject	Chemistry, Physics
Specific subject area	Spectroscopy
Type of data	Table Figure
How data were acquired	Centaur U (LTD «NanoScanTechnology», Russia) Raman spectrometer
Data format	Analyzed Filtered
Parameters for data collection	9 samples from patients with cardiovascular pathology and 11 samples from healthy patients were analyzed. Platelets were centrifuged three times at 4 °C to obtain platelet-rich plasma
Description of data collection	SERS spectra were obtained, using 532 Cobolt Samba 50 mW laser and homemade SERS substrates. Explanation of spectra has been performed using 400–1800 cm^−1^ «fingerprint» region and KnowItAll (Biorad) software.
Data source location	REC «Fundamental and Applied Photonics. Nanophotonics», Immanuel Kant Baltic Federal University, Kaliningrad, Russia
Data accessibility	With the article

**Table:** 

**Value of the data** - Raman analyzed spectra can be used for early changes and biomarkers detection in individuals with cardiovascular pathology *in vitro.* - Raman analyzed spectra can be used to reveal possible spectral differences in molecular structure for platelets in healthy individuals and individuals with cardiovascular pathology. - Raman gained data can be used as a supplementary tool in platelet spectral analysis.

## 1 Data

In this article, we present data on the human platelets SERS spectroscopy for healthy individuals and individuals with CVD. The presented data include SERS spectra for averaged and filtered data of both types (Fig. 2). SERS spectra intensities are shown on Fig. 3. The main vibrational bands are presented in Table 1. The main characteristic bands also are marked on Fig. 3. The presented data include 400–1750 cm^−1^ «fingerprint» spectral range.

**Fig. 1:**
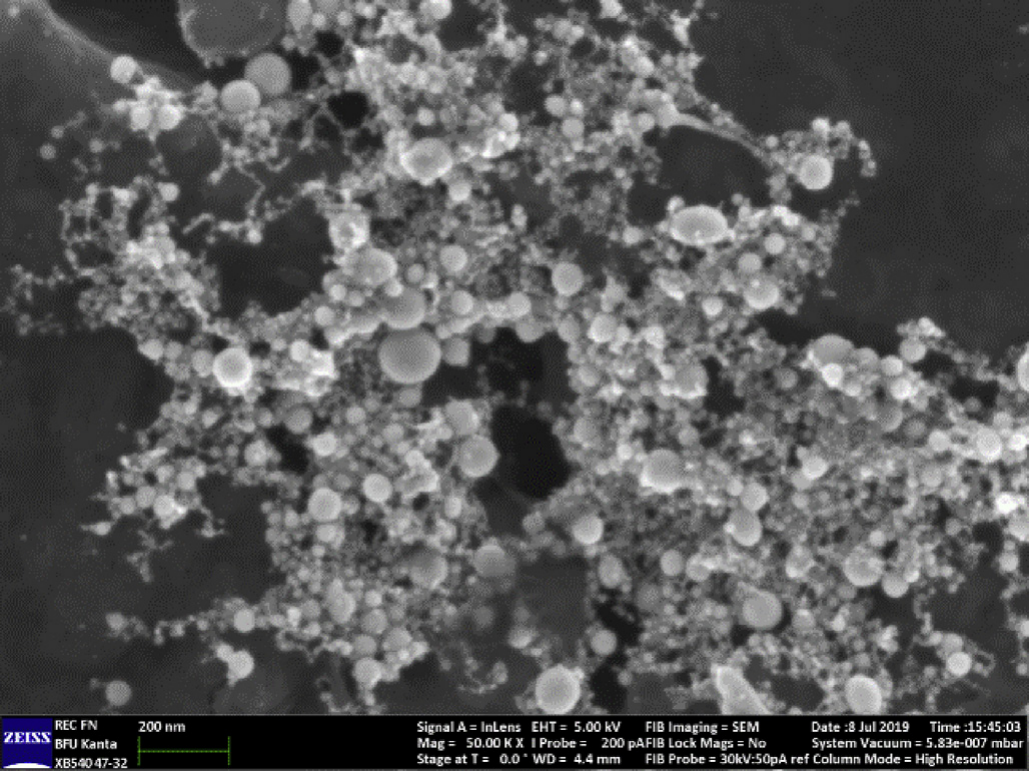
SEM image of rough titanium surface with ablative gold nanoparticles.

**Fig. 2:**
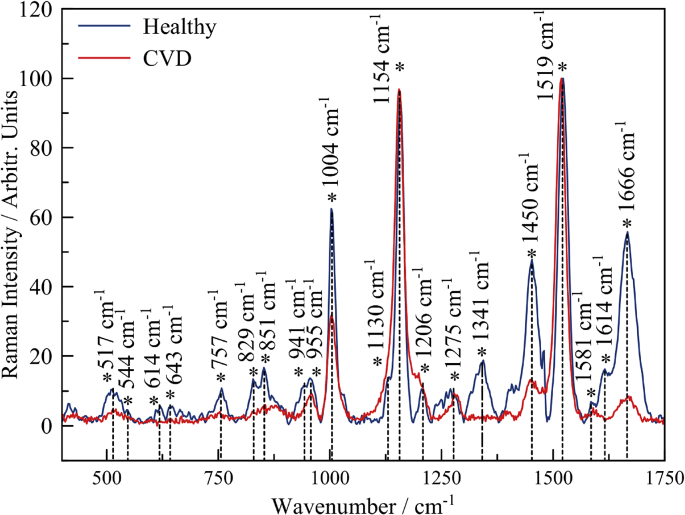
Raman spectra of platelets in 400–1750 cm^−1^ spectral region for healthy individuals (blue line) and individuals with cardiovascular pathology (red line).

**Fig. 3:**
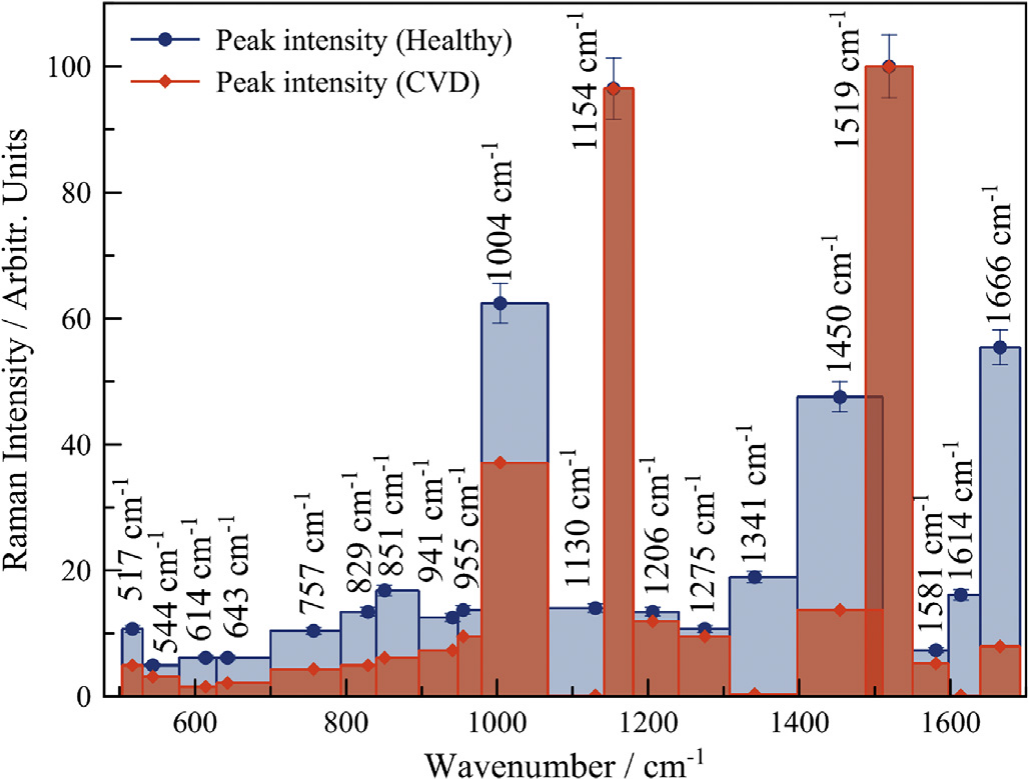
Intensity of Raman spectra for platelets for healthy individuals (blue line) and individuals with cardiovascular pathology (red line).

**Table 1:** Characteristic bands of platelets.

CCD	Healthy	Vibrational modes	Component	Reference	P-value
544 w	544 w	S–S str vibrations	Cys	[1]	P < 0.05
643 w	643 mw	C–C twist	Tyr	[1]	P < 0.05
757 w	757 mw	Phosphate diester str	Phosphatidilethanolamine	[1]	P < 0.05
829 mw	829 m	Ring br aromatic mode	Trp	[4]	P < 0.05
851 mw	851 m	Ring br aromatic mode in Tyr/phosphate groups	Proteins, phospholipids	[1]	P < 0.05
941 mw	941 m	C–C backbone vibration	Lipids, proteins	[1]	P < 0.05
955 mw	955 m	C–C backbone vibration	Lipids, proteins	[1]	P < 0.05
1004 s	1004 vs	Aromatic δ ring mode	Phe	[1]	P < 0.05
1130 m	1130 m	ν(Cβ–methyl)	proteins	[2]	P < 0.05
1154 vs	1154 vs	Bond stretching (v) C–C	proteins	[1]	P < 0.05
1275 m	1275 m	= C–H in plane deformation vibrations	Unsaturated fatty acids	[1]	P < 0.05
–	1341 m	Aromatic ring mode	Trp	[3]	P < 0.05
1450 m	1450 s	CH_2_ bend	Lipids	[1]	P < 0.05
1519 vs	1519 vs	NH3-sym bend	proteins	[4]	P < 0.05
1581 m	1581 m	Aromatic ring mode	Trp	[1]	P < 0.05
–	1614 m	Aromatic ring mode	Tyr	[1]	P < 0.05
1666 mw	1666 s	Amide I, C=C str	Proteins, cholesterol	[1]	P < 0.05

## 2 Experimental design, materials and methods

### 2.1 Subjects

11 healthy and 9 volunteers with CVD pathology were involved in study. All volunteers had signed informed consent and were approved by Immanuel Kant Baltic Federal University Independence Local Ethic Committee (Protocol № 8, May 16, 2019). All cardiological patients had a history of myocardial infarction, arterial hypertension, and were on antiplatelet therapy. Smoking patients were excluded from this study.

### 2.2 Sample preparation

Fresh venous blood samples were taken from healthy individuals and individuals with cardiovascular pathology. Blood samples were placed into centrifugal tubes containing EDTA to avoid blood coagulation. Then the fresh blood was centrifuged at 60 g for 15 min to separate platelet-rich plasma, and then the plasma at 60 g for 15 min was deposited onto the blood pellet. Platelets were finally collected by further centrifugation of the supernatant at 1500 g for 15 min. All the centrifugations were carried out at 4 °C. After platelet preparation the samples were immediately taken to be examined by SERS spectroscopy.

### 2.3 SERS substrates fabrication

Anodizing of titanium plates with a thickness of 0.1 mm was carried out on the laboratory hand-made equipment with a current source and a galvanic bath, in which titanium electrodes were immersed. An aqueous solution of KOH (5%) was used as the electrolyte. Anodizing was carried out at a current density of j = 30 mA/cm^2^ for 5 minutes. The titanium surface became a blue-colored after anodizing. Gold nanoparticles were deposited on these surfaces.

Gold nanoparticles were fabricated by the femtosecond laser ablation of gold plate in distilled water on the AVESTA unit, described in detail in Ref. [5]. The obtained nanoparticles have a plasmon resonance at λ = 530 nm.

Deposition of ablative gold nanoparticles on titanium rough surfaces was carried out by the following algorithm. First, the titanium substrate was immersed in the gold nanoparticles solution. Then the nanoparticles were deposited on the surface by evaporation of an aqueous colloidal gold solution at a temperature of 60 °C for 40 minutes.

Fig. 1 shows rough titanium surface with ablative gold nanoparticles SEM image obtained with Zeiss Cross Beam 540 electron microscope (FIB-SEM).

### 2.4 SERS experiment

SERS spectra were obtained by Centaur U («NanoScanTechnology» LTD, Russia) Raman spectrometer, using the 532 DPSS Cobolt Samba laser with 45 mW power on sample. The optical scheme included Olympus BX 41 microscope with 100X (NA 0.9) objective. Spectrometer had a focal length of 284 mm with 1200 g/mm diffraction grating and was equipped with an Andor IDus 401 CCD camera with 1024 × 256 pixels. Spectrometer had spectral resolution of 2,5 cm^−1^. The laser spot of 1 × 25 μm size was positioned at the platelets. Rayleigh scattering was eliminated by the notch filters.

Due to plasmon resonance generation availability, rough titanium surfaces with gold nanoparticles (530 nm and 570 nm for gold nanoparticles and rough Ti surface respectively) were used to enhance Raman signal up to 10^3^ times.

5 μl droplet of platelet-rich plasma was put on substrate, dried for 1 minute at room temperature, and then placed to the microscope holder. Five three times averaged spectra in different places of the droplet have been collected from each sample. Signal acquisition time was 70 s. Each time before experiment, instrument was calibrated with silicon at a static spectrum centered at 520.1 cm^−1^ for 1 s. After registration, spectra were saved as.txt and specific format (.ngs) on PC, connected to the Raman unit. KnowItAll Vibrational Spectroscopy Edition (BioRad, USA) was used for linear baseline correction and normalization. Savitsky-Golay filtering algorithm was used for all registered spectra and further analysis of peaks position and their intensity. Averaged spectra from healthy and CVD patients are displayed on Fig. 2. To determine the normal distribution for both groups (healthy and CVD samples), data were analyzed by the Student's *t*-test, and p < 0.05 was considered statistically significant with a 95% confidence interval (95%; Statistics) for the comparisons of mean Raman peaks.
